# Purification of laccase from thermophilic *Bacillus licheniformis* SO8 with three-phase partitioning, characterization, and usage in dye decolorization

**DOI:** 10.1007/s13205-026-04773-4

**Published:** 2026-05-20

**Authors:** Semih Yıldırgan, Esra Aygün, Rahime Altintas, Melda Şişecioğlu

**Affiliations:** Dept Mol Biol & Genet, Fac Sci, Ataturk University, Erzurum, Türkiye

**Keywords:** Laccase, Thermophilic *Bacillus licheniformis*, Three phase partitioning (TPP), Biochemical characterization, Decolorization

## Abstract

Laccase is an environmentally friendly biocatalyst capable of oxidizing a wide range of organic compounds and has attracted considerable interest for industrial wastewater treatment applications. In this study, laccase from the thermophilic *Bacillus licheniformis* SO8 strain (GenBank no: MG076978) was purified and characterized using the three-phase partitioning (TPP) method, a simple, rapid, low-cost, and efficient bioseparation technique, and its dye decolorization capability was preliminarily evaluated. The enzyme was purified 5.65-fold with a recovery of 102.07% under optimal TPP conditions (pH 9.0, t-butanol ratio of 1.0:1.0, and 70% ammonium sulfate), and its molecular weight was determined to be about 38.7 kDa. The purified laccase exhibited optimal activity at pH 9.0 and 70 °C and retained approximately 65% of its activity over a broad pH and temperature range. Enzyme activity was enhanced in the presence of Fe²⁺ and Mn²⁺ ions. The kinetic parameters for 2,2′-azino-bis(3-ethylbenzothiazoline-6-sulfonic acid) (ABTS) were determined as K_m_ 110 µM, V_max_ 19.6 µmol L⁻¹ min⁻¹, k_cat_ 0.048 s⁻¹, and k_cat_/Km 0.44 s⁻¹ mM⁻¹. Dye decolorization experiments indicated moderate removal efficiency, with a maximum of 38% observed for Acid Red 27, suggesting that the thermophilic laccase may serve as a potential biocatalyst for dye treatment under optimized conditions.

## Introduction

Laccase (benzenediol: oxygen oxidoreductase; EC 1.10.3.2) is a polyphenol oxidase with four copper ions in its structure. On reducing oxygen to water, these enzymes catalyze the oxidation of mono-, di- and polyphenols, aromatic compounds, organic pollutants, and inorganic substrates (Janusz et al., 2020). Due to its oxidation capabilities against a wide range of phenolic and non-phenolic compounds, laccase is an important industrial enzyme used in textiles, lignin degradation for paper production, detoxification of pollutants, wine purification, organic chemical synthesis, biosensor and cathodic reactions in enzymatic biofuel cells. The structure of the laccase contains about 15–40% carbohydrates (such as glucose, mannose, hexosamine, and arabinose) depending on the protein portion. Carbohydrates in the enzyme structure provide high stability to the laccase. The monomer mass of laccase is in the range of 50–85 kDa. Laccase, typically containing four types of copper ions per monomer, are found in various organisms such as fungi, plants, bacteria, and archaea. Bacterial laccases exhibit several advantages over those from fungal and plant sources, including broader substrate specificity, rapid and cost-effective production, a wider optimal pH range, enhanced thermostability, and tolerance to alkaline conditions (Guan et al. 2018). Despite expanding genomic knowledge of bacterial laccase diversity (Jeon and Park 2020), research on their specific properties remains limited. Previous studies have reported bacterial laccases from various strains, including *Bacillus licheniformis* LS04, *Bacillus tequilensis* SN4, *Sinorhizobium meliloti*, *Aquisalibacillus elongates*, and *Alcaligenes faecalis* (Lu et al. 2012; Sondhi et al. 2014; Pawlik et al. 2016; Rezaei et al. 2017; Mehandia et al. 2020). However, the potential of bacterial sources for laccase production remains under-explored compared to other sources (Jeon and Park 2020). Therefore, continued research into the discovery and characterization of novel bacterial laccases is crucial for expanding their biotechnological applications. While the utilization of laccase enzymes and other industrial enzymes in biotechnological processes is increasing, the substantial costs associated with large-scale enzyme isolation and purification pose a significant barrier to their widespread adoption. The main reason for these limitations lies in the fact that most purification methods are costly, time-consuming, and involve numerous steps.

As an alternative to the existing traditional methods used in the purification of laccases, three-phase separation (TPP), which is used as a simple, easy, and highly effective bioseparation technology, is widely applied by many researchers to isolate various bioactive components such as proteins, enzymes, and lipids. The economical and highly efficient TPP method consists of homogenate (bacterial culture medium), salt (usually ammonium sulfate), and an organic solvent (usually tert-butanol), and a ternary phase is observed after 1 h of incubation. The target biomolecule selectively precipitates in the interphase, while unwanted molecules accumulate in the lower aqueous phase (polar compounds) and the upper phase (non-polar compounds). By optimizing experimental conditions such as ammonium sulfate concentration, homogenate/ tert-butanol ratio, pH, and temperature, the interphase selectivity of precipitated proteins can be increased. Laccases have a strong potential in the decolorization of textile dyestuffs. Approximately 10–15% of the waste generated in the coloring of textiles is released into the environment and these substances can remain intact in the environment and cause toxic, mutagenic, and carcinogenic effects. Synthetic dyes are increasingly used in the textile industry as well as in the paper, cosmetics, and pharmaceutical industries. These compounds, chemically classified as anthraquinone, azo, heterocyclic, and tri-enylmethane dyes, cause serious environmental pollution. As an alternative to classical methods such as oxidation, chemical, adsorption, coagulation-flocculation, and electrochemical methods used for color removal, enzymatic methods are preferred today due to their potential advantages. Laccases, one of the enzymes used in enzymatically catalyzed bioremediation, can detoxify phenolic contaminants such as aromatic amines into harmLess/less harmful products through oxidative reactions (Kumar et al. 2012; Birge et al. 2022).

In this study, the purification of laccase from thermophilic *Bacillus licheniformis* SO8 strain by TPP method, which is a fast, simple, and single-step bioseparation technique, the biochemical characterization of the enzyme and the biotechnological applicability of the enzyme were investigated.

## Materials and methods

### Microorganism and chemical

In this study, *B. licheniformis* SO8 strain (GenBank number: MG076978) isolated from Nevşehir Kozaklı hot springs by Olmez et al. was used as a laccase source (Olmez, 2017). All chemical reagents and solvents used were supplied by Merck (Germany) and Sigma-Aldrich (USA).

### Enzyme and protein assay

Laccase activity was measured using a modified method adapted from Wang et al. (2014). Briefly, 0.5 mL of enzyme solution was incubated with 1 mL of 0.5 mM ABTS in 100 mM sodium phosphate buffer (pH 6.0) at 55 °C for 15 min. A control reaction was performed using buffer instead of the enzyme solution. Enzyme activity was determined spectrophotometrically at 420 nm by monitoring the oxidation of ABTS. One unit (U) of enzyme activity was defined as the amount of enzyme required to oxidize 1 µmol of ABTS per minute. Activity calculations were performed according to the formula described by Baltierra Trejo et al. (2015). Protein concentration was determined using the Bradford method with bovine serum albumin (BSA) as a standard (Bradford 1976).

### Purification of laccase

The bacterial cells present in the culture medium together with the laccase were removed by centrifugation (6000 rpm, 15 min, 4 °C). The homogenate obtained was used as the enzyme source for TPP. In the purification of laccase, firstly, to reach the highest purification efficiency, parameters such as ammonium sulfate concentration (20–80% w/v), homogenate/tert-butanol ratio (1.0:0.5, 1.0:1.0, 1.0:1.0, 1.0:1.5, 1.0:2.0 v/v) and pH (2.0–11.0) were optimized for TPP purification. In the TPP process, firstly, ammonium sulfate at different concentrations was added to 2 mL of homogenate and slowly vortexed, then t-butanol was added at different ratios and vortexed for 30 s. The mixture was incubated at room temperature for 1 h and then centrifuged at 6000 rpm for 5 min. After centrifugation, three phase formation was observed and t-butanol in the upper phase was removed and then immediately the intermediate and lower aqueous phases were collected separately in different tubes. The intermediate and lower aqueous phases were analyzed in terms of laccase activity and total protein concentration. The protein precipitate in the intermediate phase was dissolved in 0.1 M pH:7.0 sodium phosphate buffer. After determining the ammonium sulfate and t-butanol ratio, the homogenate (2 mL) was saturated with 70% ammonium sulfate and adjusted to different pH (pH 2.0–11.0) with 1 M NaOH and 1 M HCl, then t-butanol was added. The laccase activity and protein content of the lower and intermediate phases were determined as described above. Graphs representing the activity recovery (%) relative to the fold purification were plotted (Birge et al. 2023, Aras et al. 2025).

### SDS-PAGE analysis

Sodium dodecyl sulfate–polyacrylamide gel electrophoresis (SDS-PAGE) analysis was performed to determine the purity and molecular weight of partially purified laccase using the TPP method. The electrophoresis process was performed using a 12% (w/v) separating gel and a 4% (w/v) stacking gel, based on the Laemmli (1970) method. Twenty microliters of sample were loaded into each well. The gel running process was performed initially at 250 V for 5–10 min to ensure stacking phase, followed by approximately 90 min at 120 V to separate the proteins in the resolving gel. Protein bands were visualized by silver staining 20 min.). The protein molecular weight marker (ECOTECH) was used as a standard.

### Characterization of purified laccase

#### Effect of pH and temperature

To determine the optimal pH for purified laccase enzyme activity was assessed in 0.1 M buffers ranging from pH 2 to 12. Buffers used included Glycine-HCl (pH 2.0–3.0), sodium acetate (pH 4.0–5.0), phosphate buffer (pH 6.0–7.0), Tris-HCl (pH 8.0–9.0), and Glycine-NaOH (pH 10.0–11.0). To determine the optimum temperature for purified laccase, enzyme activity measurements were taken at temperatures ranging from 20 to 90⁰C.   The pH and temperature values showing the highest activity was considered 100% for relative activity calculations. To evaluate pH stability, 0.5 mL of laccase was incubated in buffers at different pH values within the indicated range for 2 h. Residual activity was then measured, with initial activity set as 100%. Thermostability was assessed by incubating the enzyme at temperatures ranging from 20 °C to 90 °C for durations of 30, 60, 90, and 120 min. Residual activity was determined at the optimal pH (pH 5.0) using 1.5 mL buffer, 1 mL substrate, and 0.5 mL enzyme solution. The initial activity was considered 100% for relative activity calculations (Akkaya et al. 2025).

#### Effect of metal ions, inhibitors, and surfactants

To investigate the effect of some metal ions on laccase, solutions of 20 mM (Li^+^, Mg^2+,^ Ca^2+^, Mn^2+^, Ni^2+^, Fe^2+^, K^+^, Zn^2+^) metal ions were used, and 0.5 mL of purified laccase was added to each metal ion to make the final concentration 1, 5 and 10 mM and incubated for 30 min, and then the remaining laccase activity was determined. As a control, the enzyme activity without any metal salt was accepted as 100% (Bilge et al. 2024; Mehandia et al. 2020). Similarly, the effect of different surfactants and inhibitors on laccase: Triton X-100, Tween-20, Tween-80 at concentrations of 1%, 5%, and 10%, SDS at concentrations of 5, 10, and 20 mM and NaF and EDTA. At the end of the incubation, the residual activity of the laccase enzyme was determined by taking the initial activity as 100%.

#### Substrate specificity

The substrate specificity of the laccase purified from the *Bacillus licheniformis* SO8 strain was determined using various phenolic (Guaiacol, Tannic acid, and L-Tyrosine) and non-phenolic (ABTS) substrates. ABTS solution of substrates was prepared in 100 mMphosphate buffer (pH: 6) and guaiacol and tannic acid in 50 mM Tris-HCl (pH 7.0) and L-tyrosine in 50 mM Tris-HCl (pH 7.0) buffers. The activity of the substrate with the highest activity of the laccase is accepted as 100 and calculated as the relative activity (%) of the other substrates.

### Enzyme kinetics

To determine the kinetic parameters ​​of laccase purified from *Bacillus licheniformis* SO8, the activities of ABTS substrate at different concentrations in the range of 50–1000 µM were measured. Lineweaver-Burk graph was drawn with the obtained values and using this graph, K_m_, V_max_, K_cat_, and catalytic activity (K_cat/_K_m_) were calculated (LaemmLi 1970; Rao et al. 2008).

### Dye decolorization

 The reaction mixture was prepared by combining 0.5 mL of the dye solution, 0.5 mL of laccase, and 1 mL of 0.5 mM ABTS mediator solution. This mixture was then incubated at 55 °C for durations of 15, 30, 60, and 120 min, with each incubation conducted at different initial pH values: 5.0, 7.0, and 9.0. The remaining dye concentrations at the end of incubation were analyzed spectrophotometrically at optimum wavelengths (Bilge et al. 2024). The percentage of color removal of the dyes was calculated according to the formula below (Rodríguez et al. 2008).

(%) Decolorization = [(Ci Ct)/Ci] x 100. Ci is the initial concentration of the dye, and Ct is the remaining dye concentration.

### Statistical analysis

All experiments involving laccase isolated from *Bacillus licheniformis* SO8 were conducted in triplicate (*n* = 3). Data are presented as mean ± standard deviation (SD) representing the average and variability of three independent experiments. Statistical analysis of laccase characterization data was performed using Student’s two-tailed t-test with GraphPad Prism^®^ software (version 9.0, GraphPad Software, San Diego, CA).

## Results and discussion

### Isolation and purification of the enzyme

**Fig. 1:**
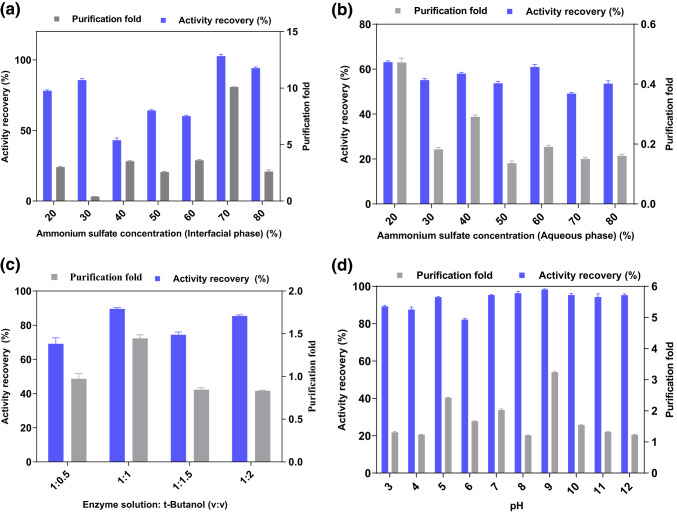
Optimization of parameters in the TPP method. **a** (NH_4_)_2_SO_4_ saturation (Interfacial phase) (%); **b** (NH_4_)_2_SO_4_ saturation (Aqueous phase) (%), **c** Enzyme solution: t-butanol ratio; **d** pH effect at 70% (w/v) (NH_4_)_2_SO_4_ saturation

To obtain laccase resistant to extreme conditions; *Bacillus licheniformis* SO8 bacteria, which has the highest activity among the thermotolerant bacteria previously isolated and identified in our laboratory, was selected.t. TPP, a simple, rapid, and one-step bioseparation technique, was used to purify laccase from liquid cultures of bacteria as an alternative to expensive, time-consuming, and multi-step traditional methods. First of all, to purify the laccase isolated from *Bacillus licheniformis* SO8 strain by TPP method with the highest yield, parameters such as t- butanol, ammonium sulfate, pH, and ratio were optimized. Optimizing purification conditions is crucial for achieving high enzyme purity and yield (Kumar et al., 2011). Figure 1 illustrates the effects of various parameters on the purification coefficient and yield of laccase from the homogenate. Optimal conditions were determined to be 70% (w/v) t-butanol concentration, 1.0:1.0 homogenate solution: t-butanol ratio, and pH 9.0, resulting in a 5.65-fold purification and 102.07% yield (Fig. 1).

 Table 1 summarizes the overall purification of SO8 laccase using TPP. Under optimized conditions, TPP effectively purified laccase from the *Bacillus licheniformis* SO8 crude extract, achieving a 102.07% yield and 5.65-fold purification. An increase in t-butanol concentration leads to increased water uptake into the organic phase, elevating the aqueous phase salinity and consequently protein precipitation at the interface (Zouari-Mechichi et al. 2006). Laccases typically partition to the interface phase during TPP (Kumar et al. 2012; Altıntas et al. 2025). The yield exceeding 100% is a result of the TPP method’s capacity to remove inhibitors and activate the enzyme during purification. Additionally, the combined effect of ammonium sulfate and t-butanol used during the TPP process can cause positive changes in the enzyme’s three-dimensional conformation, enabling it to transition to a more active structure. Previous studies have demonstrated the suitability of TPP for laccase purification with high yields. For example, a study reported 27.8-fold purification and 161% yield for laccase from *Pleurotus ostreatus* (Reference not specified). Rajeeva and Lele (2011) achieved 60% yield and 13.1-fold purity for Ganoderma sp. laccase using two-stage TPP. Liu et al. reported 20-fold purity and approximately 75% yield for laccase purified by TPP. When compared to other commonly used methods such as ammonium sulfate precipitation-immobilized metal affinity chromatography (46-fold purification, 57.4% yield) for *Pleurotus ostreatus* laccase (do Rosário Freixo et al. 2012), diafiltration (3.7-fold purification, 90.4% yield) for *Ganoderma* sp. laccase (Rajeeva and Lele 2011), and ultrafiltration (2.14-fold purification, 87% yield) for *Pycnoporus* sp. laccase (Lu et al. 2007), TPP often demonstrates superior purification yields. TPP is an emerging bioseparation technique for protein precipitation, enabling efficient enzyme purification and recovery (Dennison and Lovrien 1997; Kumar et al. 2012). Compared to traditional chromatographic methods, TPP has shown potential for achieving higher purity for enzymes such as protease and amylase (Saxena et al., 2007). TPP is increasingly recognized for its versatility and is now widely employed in various industrial sectors for protein recovery (Kumar et al. 2012).

**Table 1:** Purification table of laccase purified from *B. licheniformis* SO8

_Purification steps_		_Total activity_ _(EU/mL)_	_Total Protein (mg/mL)_	_Specific activity (EU/mg)_	_Recovery %_	_Purification fold_
_Crude extract_		_72.446_	_0.520_	_139.32_	_100_	_1_
_TPP- interfacial phase_		_73.948_	_0.094_	_786.68_	_102.07_	_5.65_
_TPP – aqueous phase_		_31.08_	_0.256_	_121.4_	_42.9_	_0.87_

**Table 2:** Effect of some surfactants and inhibitors

Control	Concentration	Residual Activity (%)
	-	100
*Surfactants (% v/v)*		
TritonX-100	1	82.26 ± 2.652
	5	41.72 ± 5.855
	10	19.92 ± 0.489
Tween-20	1	100.3 ± 1.495
	5	98.39 ± 7.159
	10	40.79 ± 0.233
Tween-80	1	119.9 ± 18.87
	5	103.2 ± 11.36
	10	41.72 ± 3.527
*Inhibitors (mM)*		
SDS	5	100.8 ± 3.604
	10	92.83 ± 4.404
	20	58.97 ± 8.414
NaF	5	90.54 ± 3.871
	10	79.93 ± 0.998
	20	60.61 ± 8.159
EDTA	5	114.1 ± 13.40
	10	82.97 ± 1.710
	20	49.65 ± 1.456

### SDS-PAGE analysis

**Fig. 2:**
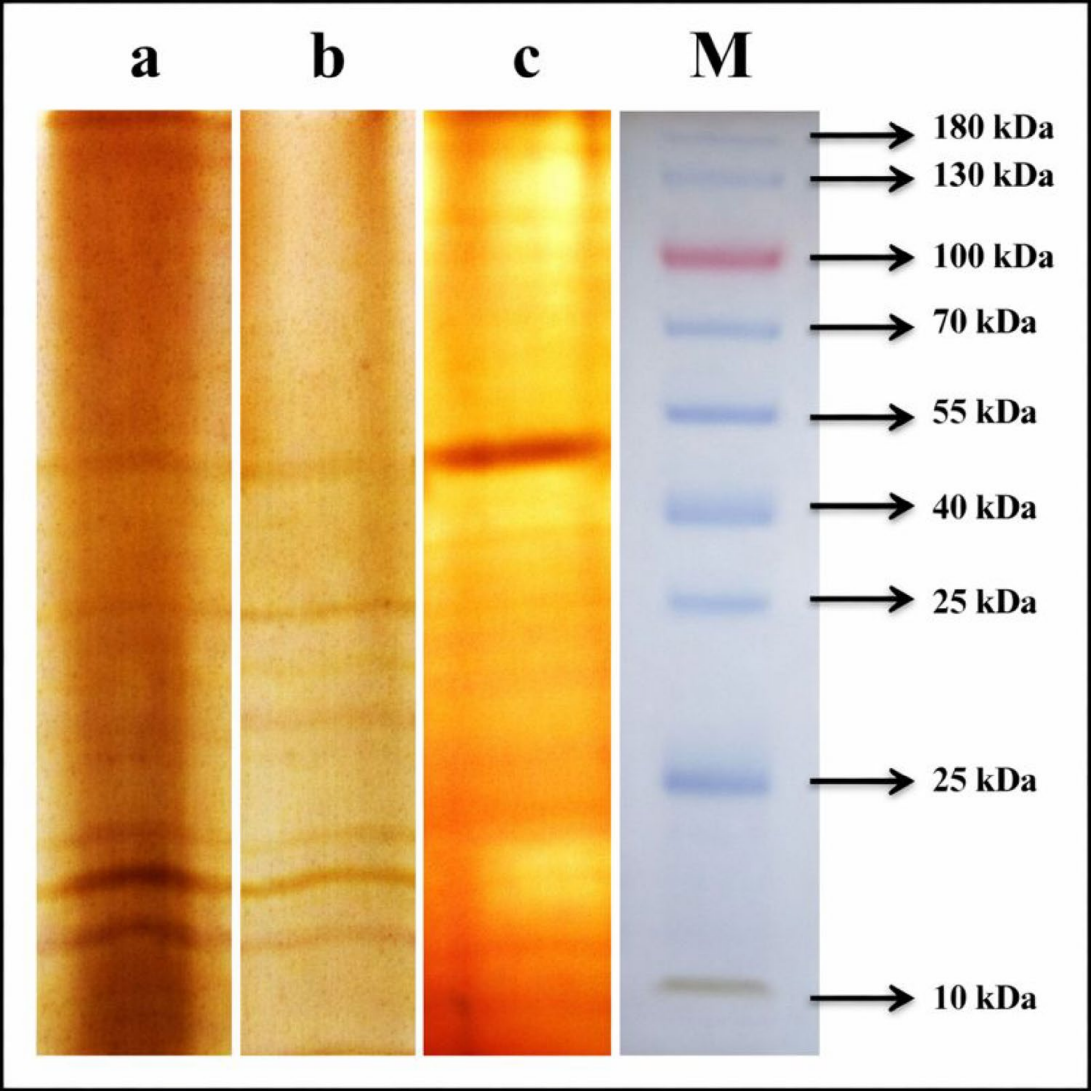
SDS-PAGE analysis of laccase from *B. licheniformis* SO8 . M: markers; **a** Crude extract; **b** Aqueous phase; **c** Interfacial precipitate

SDS-PAGE analysis confirmed the efficacy of two-phase partitioning (TPP) for laccase purification (Fig. 2). A single protein band was observed exclusively in the interphase (lane c), in contrast to the crude enzyme extract (lane a) and the aqueous phase (lane b). This observation strongly suggests that TPP effectively removes contaminating proteins and other molecules, enriching the interphase with the target laccase enzyme. The molecular weight of the purified *Bacillus licheniformis* SO8 laccase was determined to be approximately 38.7 kDa based on the SDS-PAGE analysis. This molecular weight falls within the range reported for laccases from other bacterial species, such as 32 kDa for *B. tequilensis* SN4 laccase (Sondhi et al. 2014), 50.11 kDa for *Enterococcus faecium* A2 laccase (Birge et al. 2022), and 160 kDa for *Arthrobacter gonensis* P39 laccase (Yanmış et al. 2016).

### Effect of pH and temperature on enzyme activity and stability

**Fig. 3:**
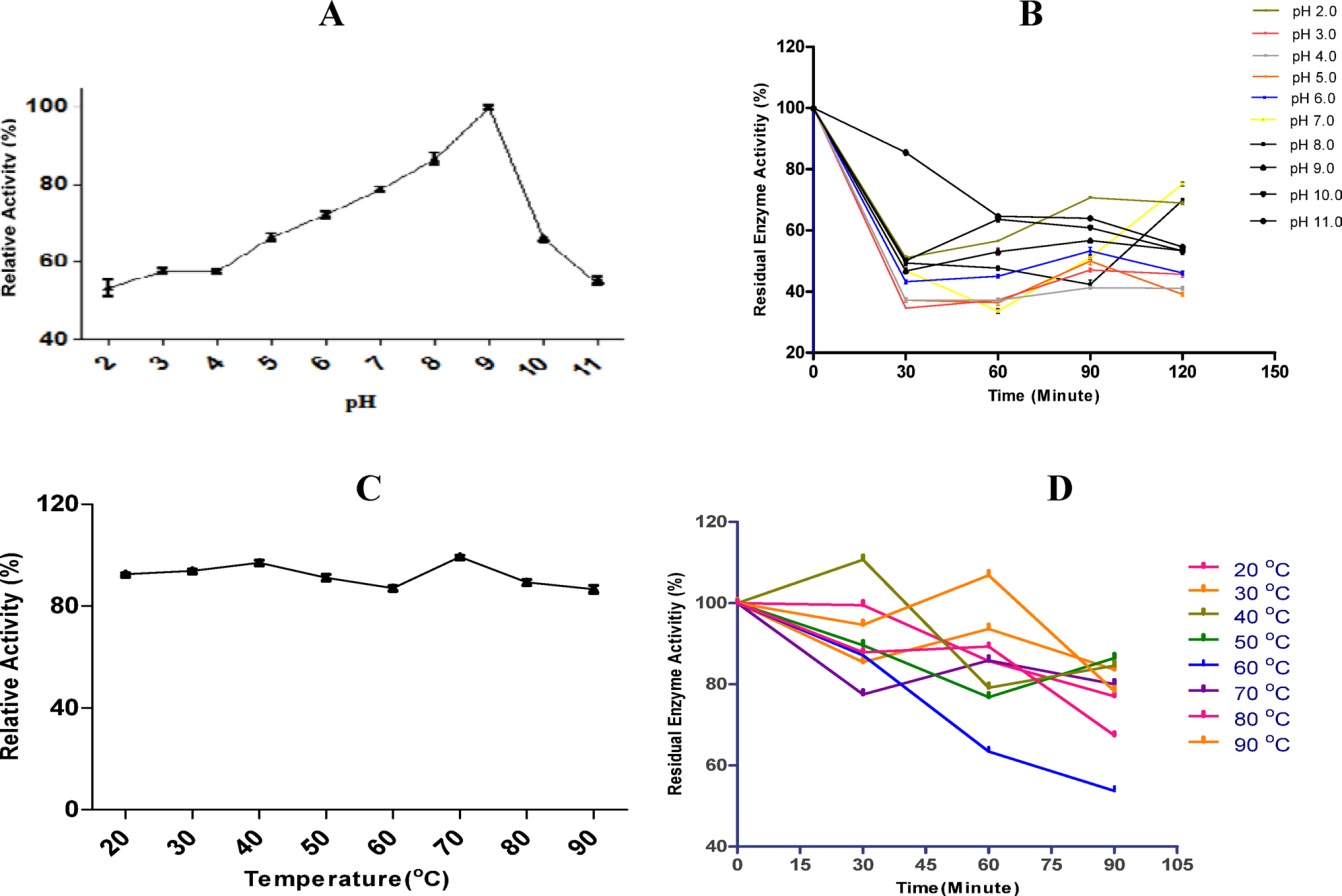
(**A**–**B**) Effect of pH on the relative activity (**A**) and stability (**B**) of purified laccase. While the effect of pH on relative activity, the laccase was incubated in four different buffer systems (Glycine – HCl buffer at pH 2.0-3.0, sodium acetate buffer at pH 4.0–5.0, sodium phosphate buffer at pH 6.0–7.0, Tris-HCl buffer at pH 8.0–9.0 and Glycine-NaOH at pH:10–11) . pH stability was investigated by incubating the purified enzyme in respective buffers (50 mM) in the pH range from 2.0 to 11.0 at room temperature for 30–120 min. **(C-D)** Effect of temperature on the relative activity (**C**) and stability (D) of purified laccase. Relative activity of the laccase was investigated in the temperature range from 20 to 90^◦^C at optimal pH (in Tris-HCl buffer at pH 9.0). The effect of temperature on the stability of purified laccase was examined at 20–90^◦^C at optimal pH (in Tris-HCl buffer at pH 9.0) for 30–90 min

The optimum pH of the laccase enzyme purified from *B. licheniformis* strain SO8 was determined to be 9.0, indicating that it is an alkaline enzyme (Fig. 3A). Laccase enzyme was found to retain over 50% activity over a wide pH range (pH 2.0–11.0) (Fig. 3B). It was observed that the laccase isolated from strain SO8 remained stable at high pH values and retained over 35% of its activity at the end of 120 min at all pH values. Laccases that show activity and remain stable at high pH values are highly demanded industrially. While a decrease in relative activity is observed at 60 min, the recovery and increase in relative activity at 120 min is indicative of the enzyme’s resistance to extreme conditions, representing a structural recovery and activation process following the initial environmental stress. Alkali-resistant laccase from *Streptomyces and Thioalkalivibrio* (Machczynski et al. 2004; Lu et al. 2012; Ausec et al. 2015; Ruijssenaars and Hartmans 2004), most originate from *Bacillus species*. To determine the temperature profile and stability of *B. licheniformis* SO8 laccase enzyme, it was studied at temperatures between 20 °C and 90 °C and showed maximum activity at 70 °C and high activity at 20–90 °C (Fig. 3C). The activity measurements of the laccase enzyme were carried out at temperatures between 20 °C and 90 °C for 120 min at periodic intervals. It was observed that the laccase retained its activity significantly during the 2 h incubation period at all temperatures studied (Fig. 3D). The high heat resistance of laccases is a result of their evolutionary adaptations, which they have gained thanks to the dense carbohydrate groups they contain, their strong secondary structure bonds, and especially their bacterial origin. It mustn’t lose its activity for a long time at these temperature ranges because especially the wastewater from textile factories can be at high temperatures. Cooling water demands both time and resources. Prior studies have demonstrated significant thermal instability in laccases from various sources. For instance, Koschorreck et al. (2008) reported a 92% activity loss for a fungal laccase after 2 h at 70 °C, while Halaburgi et al. (2011) observed a 52% activity loss in *C. cladosporioides* laccase within 5 min at 80 °C. In contrast, the *B. licheniformis* SO8 laccase exhibited exceptional thermostability. This remarkable stability suggests a high tolerance to elevated temperatures and a broad operational temperature range.

###  Effect of metal ions, inhibitors and surfactants

**Fig. 4:**
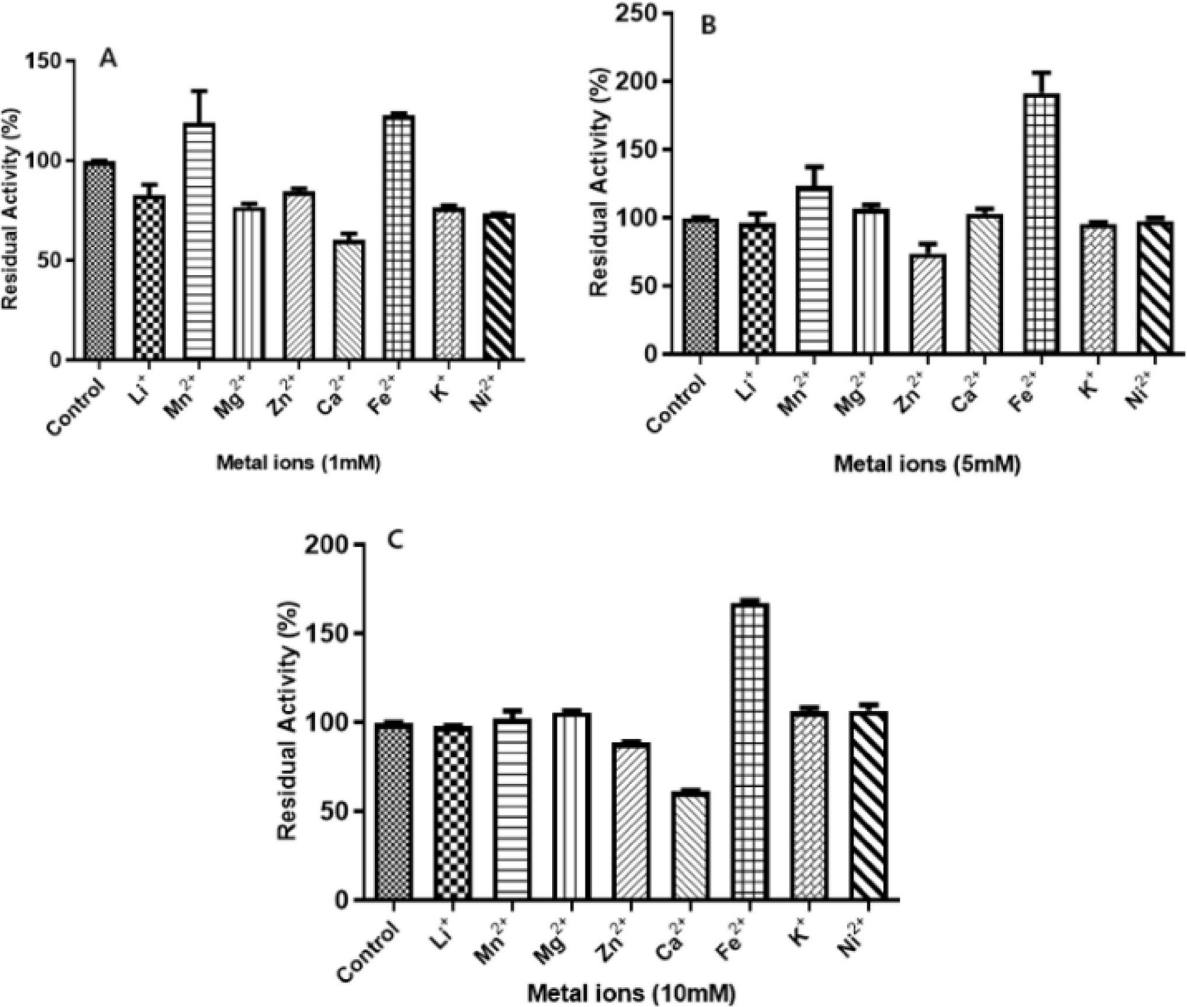
Effect of metal ions (1 mM, 5 mM and 10 mM), on the relative activity of purified laccase. The effects of metal ions (Li^+^, Mn^2+^ Mg^2+^, Zn^2+^, Ca^2+^, Fe2^+^, Ni^2+^ and K^+^) on the laccase activity were tested by incubating the laccase in the presence of any of mineral salts (CaCl2, MgSO4, FeCl3, MnSO4 and CuSO4) at the different concentrations from 1, 5 and 10 mM for 30 min

The effect of various metal ions (Li^+^, Mg^2+,^ Ca^2+^, Mn^2+^, Ni^2+^, Fe^2+^, K^+^, Zn^2+^) on *Bacillus licheniformis* SO8 laccase activity was investigated. . At concentrations of 1 and 5 mM of Fe^2+^ and Mn^2+^ ions, the activity of the laccase enzyme was increased. In the presence of other metal ions, enzyme activity was conserved by more than 50% (Fig. 4A and B). At a concentration of 10 mM Ca^2+^ and Zn^2+^ ions, the enzyme was observed to retain 61% and 88% of its activity, respectively However, 67% increase in enzyme activity was observed in the presence of Fe^2+^ ions, while the activity remained constant in the presence of other metal ions (Fig. 4C). ^2+^ Among laccases from other sources (Lu et al., 2013; Zhu et al., 2011), the laccase produced by *Bacillus licheniformis* SO8 demonstrates remarkable tolerance to common environmental metal ions. This is a critical advantage for industrial applications, as high concentrations of various metals often encountered in industrial settings can significantly inhibit laccase activity. Consequently, developing laccases with intrinsic resistance to these metal ions is highly desirable for their successful integration into industrial processes (Sondhi et al. 2014). The Triton X-100, Tween-80, and Tween-20 at 1%, 5%, and 10% concentrations; SDS, NaF, and EDTA at 5, 10, and 20 mM concentrations were used to investigate the effects of surfactants and some chemicals on *B. licheniformis* SO8 laccase enzyme activity. Triton X-100 showed the highest inhibition effect at 10% concentration and inhibited the enzyme by 81%. At concentrations of 10% and 20 mM, Triton x-100 and EDTA were the most inhibitory molecules. At concentrations of 10 and 20 mM, the decrease in enzyme activity in the presence of NaF, and EDTA, was 21%, 40%, 17%, and 51%, respectively. The *B. licheniformis* SO8 laccase demonstrated high stability at 5 mM and 1% concentrations of all tested surfactants and chemicals. Notably, its activity was enhanced by EDTA (5mM), and Tween-80 (1 and 5%) (Table 2). While ionic surfactants often inhibit laccase activity (Zhang et al. 2013), some studies have reported stimulation by surfactants (Diamantidis 2000, Sondhi 2014, Lu 2013). Similarly, the beneficial effects of non-ionic surfactants like Triton X-100 (Ji GuangLei et al. 2009, Gu et al. 2014) and the stability of laccases in the presence of Tween-20 and Tween-80 (Sondhi 2014) have been previously documented. This robust stability suggests potential applications for the *B. licheniformis* SO8 laccase in treating industrial waste, particularly in dye removal from textile effluents.

Furthermore, the lack of significant inhibition by EDTA aligns with previous findings (Lu et al., 2013), likely due to the limited accessibility of EDTA to the enzyme’s copper atoms (Safary et al. 2016). While fluoride ions are generally potent laccase inhibitors (Dube et al. 2008), their inhibitory effects are pH-dependent. This observation is consistent with the stability of the *B. licheniformis* SO8 laccase and the *Streptomyces viridochromogenes* recombinant laccase, both of which exhibit activity and stability at alkaline pH (Trubitsina et al. 2015).

**Substrate specificity and kinetic study** ABTS, guaiacol, tannic acid and L-tyrosine substrates were used to determine the specificity of the laccase enzyme purified from *B. licheniformis* SO8 for various phenolic and non-phenolic substrates. Laccase enzyme showed the highest activity for ABTS substrate. The specificity of the enzyme for other substrates was determined as guaiacol (60%), tannic acid (52%) and L-tyrosine (9%), respectively (Table 3). The ability of *B. licheniformis* SO8 laccase to oxidize ABTS and its low specificity for tyrosine indicate that it is a true laccase, in agreement with studies in the literature (Sondhi et al. 2015).

**Table 3:** Substrate specificity

_Natural substrates_	_pH_	_ʎ(nm)_	_Relative Activity(%)_
_ABTS_	_6.0_	_420_	_100.0 ± 0.001_
_Guaiakol_	_8.0_	_470_	_60.20 ± 2.427_
_Tannik asit_	_7.0_	_250_	_52.97 ± 1.784_
_L-tirozin_	_7.0_	_280_	_9.200 ± 0.642_

**Fig. 5:**
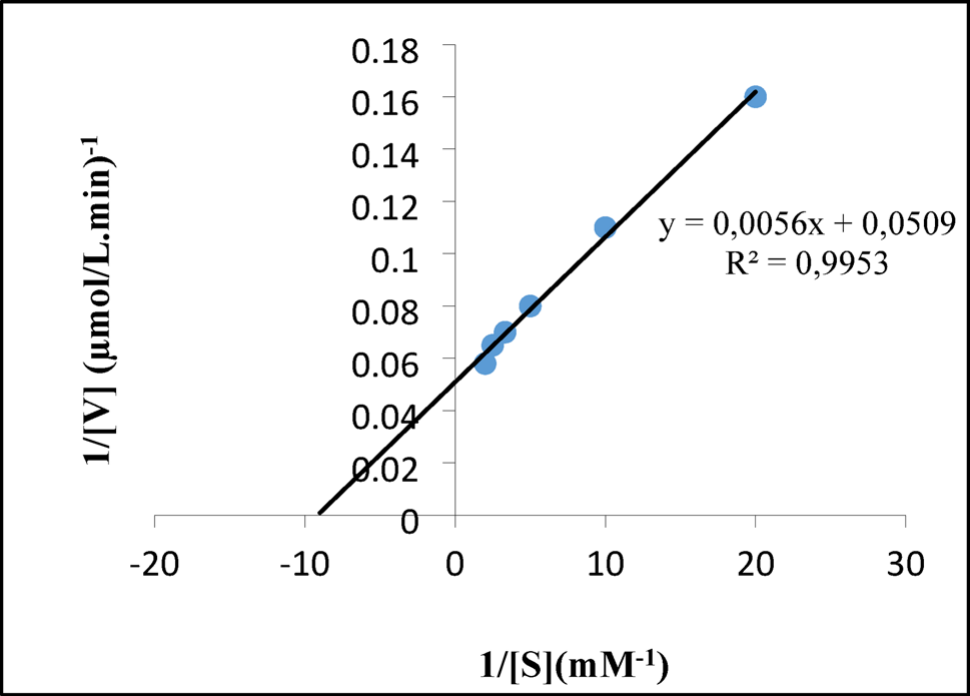
Lineweaver-Burk plot for K_m_ and V_max_ for ABTS substrate

Kinetic studies were carried out using the laccase enzyme obtained from *B. licheniformis* SO8 bacteria. The Km value obtained for the ABTS substrate of the laccase enzyme was 110 µM, Vmax value was 19.6 µmol.L ^-^^1^.min^-1^, K_cat_ value was 0.048 s^-1^ and K_cat_/K_m_ value was 0.44 s^-1^. mM^-1^*. Bacillus* sp. HR03 (K_m_ value 0.535 mM) (Mohammadian 2010), *Bacillus tequilensis* SN4 (Km value 1.404 ± 0.08 mM) (Sondhi 2014), *Alcaligenes faecalis* (Km value 500 µM) (Mehandia et al. 2020) and *Thioalkalivibrio sp.* (Km value 4.6 ± 1.1 mM) (Ausec 2015, *B. licheniformis* SO8 laccase has a higher affinity for ABTS substrate. However, it is known that the oxidation efficiency and substrate affinity of laccases depend significantly on the substitution and nature of the phenolic ring and the type of substrate used (Mohammadian et al. 2010) (Fig. 5).

### Decolorization

**Fig. 6:**
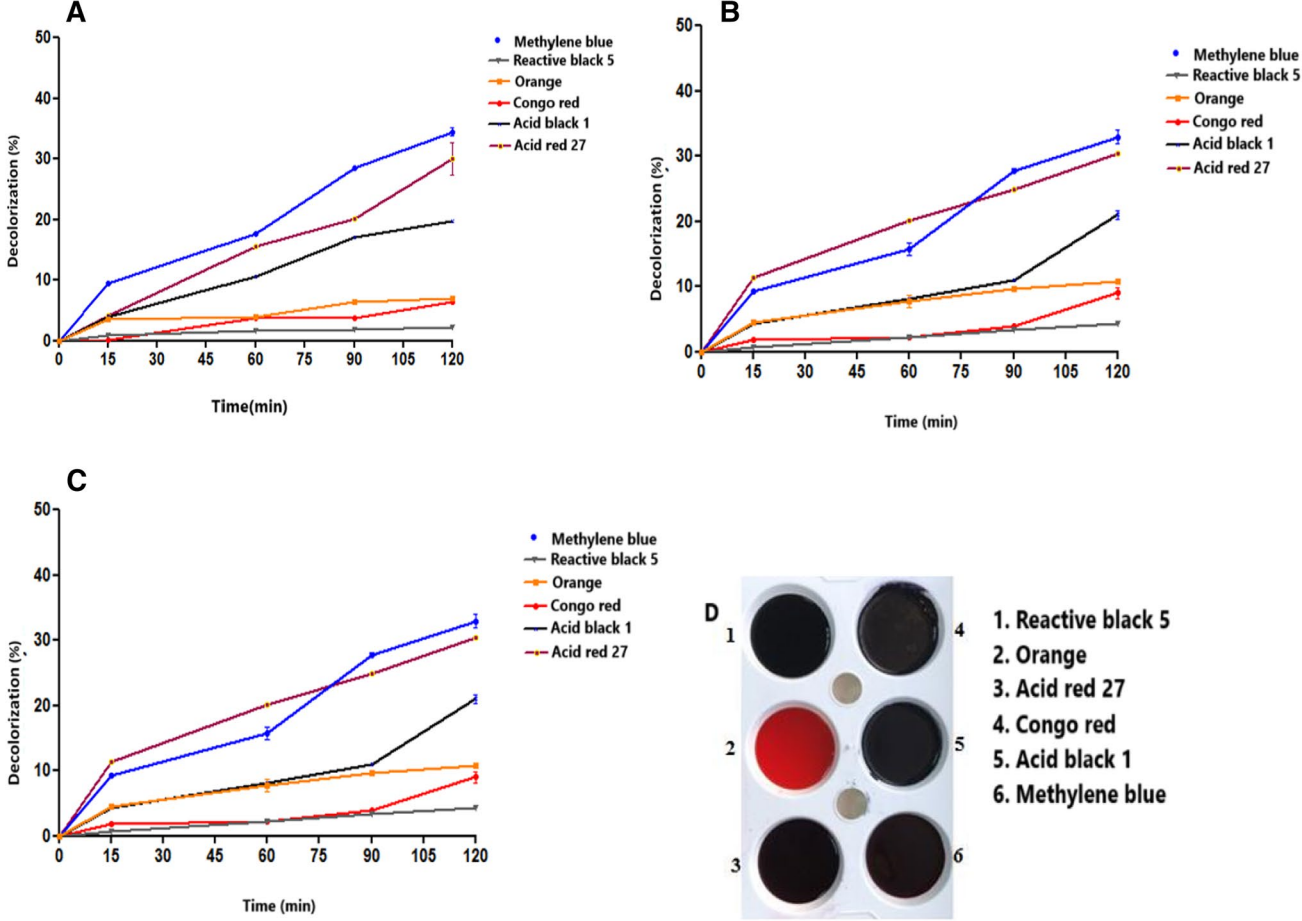
Color removal of six different dyes during 120 min of incubation at (a) pH 5, (b) pH 7, and (c) pH 9

The decolorization process with *B. licheniformis* SO8 laccase was carried out in the presence of 0.5 mM ABTS to enhance the oxidative effect of the enzyme (Fig. 6). The decolorization of the azo dyes tested with *B. licheniformis* SO8 laccase purified under all conditions studied (pH 5.0- pH 7.0- pH 9.0 at 55 °C for 15, 30, 60, and 120 min) was determined to be approximately 1–40%. At the end of 120 min at pH 9.0, the laccase enzyme provided the best decolorization of acid red 27 at 37.467%, methylene blue at 36.68%, and acid black 1 at 22.3%. Reactive black 5, Congo red and Orange dyes did not show much effect. When the results were evaluated, it was determined that the highest dye removal was 37.467% for acid red 27 dye at pH 9.0. As the incubation time increased, it was observed that the percentage removal of the colors of the dyes was also higher. Dye decolorization is a prominent biotechnological application of bacterial laccases. Many dyes, particularly azo and anthraquinone dyes, possess large molecular sizes or high redox potentials, rendering them inaccessible for direct oxidation by laccase. To overcome this limitation, “electron shuttles” or mediators are employed. 2,2’-Azino-bis(3-ethylbenzthiazoline-6-sulfonic acid) (ABTS) was the first synthetic mediator identified to enhance laccase activity. ABTS has proven to be an effective mediator in dye decolorization, significantly improving laccase performance in this field (Guan, 2018; Jeon and Park 2020). When looking at similar studies; Birge et al. (2022) recorded that the laccase isolated from *Enterococcus faecium* A2 achieved the highest dye removal rate of 40% on Acid Red 27 and 36% on Reactive Black 5. In another study, Mehandia et al. (2020) reported that the dye removal efficiency of the laccase isolated from *Alcaligenes faecalis* XF1 reached 88% for Indigo Carmine and 77% for Malachite Green in the presence of HBT mediator (Birge et al. 2022; Mehandia et al. 2020). Based on these results, the dye removal efficiency of laccase was found to be relatively lower compared to some previously reported studies. This may be attributed to the high redox potential and large molecular structure of the dyes, which can limit their accessibility to the enzyme’s active site, as well as the short incubation period that may have prevented the full manifestation of the enzyme’s oxidative activity. Nevertheless, these findings suggest that laccase has the potential to be an effective biocatalyst for dye removal, particularly if reaction conditions are optimized and longer incubation times are applied.

## Conclusion

Despite advancements in biotechnology, the commercialization of laccases is hindered by the complexity and cost of large-scale enzyme purification. Traditional purification methods often pose significant challenges. This study successfully employed a novel approach, Three-phase partitioning (TPP), for the single-step purification of laccase from *Bacillus licheniformis* SO8. TPP proved to be a rapid, economical, and efficient alternative, achieving a 5.65-fold purification with 102% yield. The purified laccase exhibited remarkable stability across a broad pH range (2.0–11.0) and temperature range (20–90 °C). Notably, it retained 92% of its activity at elevated temperatures, surpassing the thermostability of many bacterial laccases reported in the literature. Moreover, the enzyme demonstrated significant tolerance to a range of metal ions (Li^+^, Mg^2+^, Ca^2+^, Mn^2+^, Ni^2+^, Fe^2+^, K^+^, Zn^2+^), chemicals/inhibitors (NaF and EDTA), and surfactants (SDS, Triton X-100, Tween-20, Tween-80). This robust stability, combined with its broad activity spectrum and decolorization potential, positions *B. licheniformis* SO8 laccase as a promising candidate for diverse industrial applications, particularly in bioremediation.

## References

[CR1] Akkaya SN, Almansour A, Altintas R, Sisecioglu M, Adiguzel A (2025) Purification, characterization, optimization, and docking simulation of alkaline protease produced by *Brevibacillus agri* SAR25 using fish wastes as a substrate. Food Chem 142816. 10.1016/j.foodchem.2025.142816 10.1016/j.foodchem.2025.142816 39798358

[CR2] Altıntas R, Güngör S, Aygün E, Bakan B, Şişecioğlu M (2025) Immobilization of L-glutaminase from *Bacillus arachidis* E12 on glutamic acid activated aluminum oxide nanoparticles (Al2O3 NPs): Purification, biochemical characterization, and anticancer activity. Int J Biol Macromol, 146731 10.1016/j.ijbiomac.2025.146731 40789406

[CR3] Aras S, Altıntas R, Aygun E, Goren G, Sisecioglu M (2025) Purification of α-amylase from thermophilic *Bacillus licheniformis* SO5 by using a novel method, alternating current magnetic-field assisted three-phase partitioning: Molecular docking and bread quality improvement. Food Chem 484:144258. 10.1016/j.foodchem.2025.144258 40286717 10.1016/j.foodchem.2025.144258

[CR4] Ausec L, Črnigoj M, Šnajder M, Ulrih NP, Mandic-Mulec I (2015) Characterization of a novel high-pH-tolerant laccase-like multicopper oxidase and its sequence diversity in *Thioalkalivibrio sp*. Appl Microbiol Biotechnol 99:9987–9999. 10.1007/s00253-015-6843-3 26227413 10.1007/s00253-015-6843-3

[CR5] Birge A, Alcicek EA, Baltaci MO, Sisecioglu M, Adiguzel A (2022) Purification and biochemical characterization of a new thermostable laccase from *Enterococcus faecium* A2 by a three-phase partitioning method and investigation of its decolorization potential. Arch Microbiol 204(8):533. 10.1007/s00203-022-03054-x 35906438 10.1007/s00203-022-03054-x

[CR500] Bilge S, Dogan-Topal B, Gürbüz MM, Ozkan SA, Sınağ A (2024) Recent trends in core/shell nanoparticles: their enzyme-based electrochemical biosensor applications. Microchimica Acta 191(5):240 10.1007/s00604-024-06305-4 PMC10994877 38573400

[CR6] Bradford MM (1976) A rapid and sensitive method for the quantitation of microgram quantities of protein utilizing the principle of protein-dye binding. Anal Biochem 72(1–2):248–254. 10.1016/0003-2697(76)90527-3 942051 10.1016/0003-2697(76)90527-3

[CR7] Dennison C, Lovrien R (1997) Three phase partitioning: concentration and purification of proteins. Protein Exp Purif 11(2):149–161. 10.1006/prep.1997.0779 10.1006/prep.1997.0779 9367811

[CR8] Diamantidis G, Effosse A, Potier P, Bally R (2000) Purification and characterization of the first bacterial laccase in the rhizospheric bacterium *Azospirillum lipoferum*. Soil Biol Biochem 32(7):919–927. 10.1016/S0038-0717(99)00221-7

[CR9] do Rosário Freixo M, Karmali A, Arteiro JM (2012) Production, purification, and characterization of laccase from *Pleurotus ostreatus* grown on tomato pomace. World J Microbiol Biotechnol 28:245–254. 10.1007/s11274-011-0813-4 22806800 10.1007/s11274-011-0813-4

[CR10] Dube E, Shareck F, Hurtubise Y, Daneault C, Beauregard M (2008) Homologous cloning, expression, and characterisation of a laccase from *Streptomyces coelicolor* and enzymatic decolourisation of an indigo dye. Appl Microbiol Biotechnol 79:597–603. 10.1007/s00253-008-1475-5 18437373 10.1007/s00253-008-1475-5

[CR11] Gu C, Zheng F, Long L, Wang J, Ding S (2014) Engineering the Expression and Characterization of Two Novel Laccase Isoenzymes from *Coprinus comatus* in *Pichia pastoris* by Fusing an Additional Ten Amino Acids Tag at N-Terminus. PLoS ONE 9(4):e93912. 10.1371/journal.pone.0093912 24710109 10.1371/journal.pone.0093912 PMC3977997

[CR12] Guan ZB, Luo Q, Wang HR, Chen Y, Liao XR (2018) Bacterial laccases: promising biological green tools for industrial applications. Cell Mol Life Sci 75:3569–3592. 10.1007/s00018-018-2883-z 30046841 10.1007/s00018-018-2883-z PMC11105425

[CR13] Halaburgi VM, Sharma S, Sinha M, Singh TP, Karegoudar TB (2011) Purification and characterization of a thermostable laccase from the ascomycetes *Cladosporium cladosporioides* and its applications. Process Biochem 46(5):1146–1152. 10.1016/j.procbio.2011.02.002

[CR14] Janusz G, Pawlik A, Swiderska-Burek U, Polak J Justyna Sulej, Anna Jarosz Wilkołazka and Andrzej Paszczynski., 2020, Laccase Properties, Physiological Functions, and Evolution. Int J Mol Sci 21:966. 10.3390/ijms21030966 10.3390/ijms21030966 PMC7036934 32024019

[CR15] Jeon SJ, Park JH (2020) Refolding, characterization, and dye decolorization ability of a highly thermostable laccase from *Geobacillus sp.* JS12. Protein Exp Purif 173:105646. 10.1016/j.pep.2020.105646 10.1016/j.pep.2020.105646 32315700

[CR16] Ji GuangLei JG, Zhang HaiBo ZH, Huang Feng HF, Huang XiRong HX (2009) Effects of nonionic surfactant Triton X-100 on the laccase-catalyzed conversion of bisphenol A. 10.1016/S1001-0742(08)62444-4 10.1016/s1001-0742(08)62444-4 20108679

[CR17] Kesebir AÖ, Kılıç D, Şişecioğlu M, Adıgüzel A, Küfrevioğlu Öİ (2021) Recombinant laccase production from *Bacillus licheniformis* O12: Characterization and its application for dye decolorization. Biologia 76(11):3429–3438. 10.1007/s11756-021-00847-1

[CR18] Koschorreck K, Richter SM, Swierczek A, Beifuss U, Schmid RD, Urlacher VB (2008) Comparative characterization of four laccases from *Trametes versicolor* concerning phenolic C–C coupling and oxidation of PAHs. Arch Biochem Biophys 474(1):213–219. 10.1016/j.abb.2008.03.009 18367094 10.1016/j.abb.2008.03.009

[CR510] Kumar NJI, Verma Y, Kumar RN (2011) Spatial analysis of composition and species interactions with temporal variation of zooplankton community of shallow tropical lake: Thol bird sanctuary, India. Univ J Environ Res Technol 1(2):151–159

[CR19] Kumar VV, Kumar MP, Thiruvenkadaravi KV, Baskaralingam P, Kumar PS, Sivanesan S (2012) Preparation and characterization of porous cross linked laccase aggregates for the decolorization of triphenyl methane and reactive dyes. Bioresour Technol 119:28–34. 10.1016/j.biortech.2012.05.078 22728178 10.1016/j.biortech.2012.05.078

[CR20] Laemmli UK (1970) Cleavage of structural proteins during the assembly of the head of bacteriophage T4. *nature*, *227*(5259), 680–685. 10.1016/0022-2836(70)90379-7 10.1038/227680a0 5432063

[CR21] Lu L, Zhao M, Wang Y (2007) Immobilization of laccase by alginate–chitosan microcapsules and its use in dye decolorization. World J Microbiol Biotechnol 23:159–166. 10.1007/s11274-006-9205-6

[CR22] Lu L, Zhao M, Wang TN, Zhao LY, Du MH, Li TL, Li DB (2012) Characterization and dye decolorization ability of an alkaline resistant and organic solvents tolerant laccase from *Bacillus licheniformis* LS04. Bioresour Technol 115:35–40. 10.1016/j.biortech.2011.07.111 21868217 10.1016/j.biortech.2011.07.111

[CR601] Lu L, Wang TN, Xu TF, Wang JY, Wang CL, Zhao M (2013) Cloning and expression of thermo-alkali-stable laccase of *Bacillus licheniformis* in Pichia pastoris and its characterization. Biores Technol 134:81–86 10.1016/j.biortech.2013.02.015 23500563

[CR23] Machczynski MC, Vijgenboom E, Samyn B, Canters GW (2004) Characterization of SLAC: a small laccase from *Streptomyces coelicolor* with unprecedented activity. Protein Sci 13(9):2388–2397. 10.1110/ps.04759104 15295117 10.1110/ps.04759104 PMC2280001

[CR24] Mehandia S, Sharma SC, Arya SK (2020) Immobilization of laccase on chitosan-clay composite beads to improve its catalytic efficiency to degrade industrial dyes. Mater Today Commun 25:101513. 10.1016/j.mtcomm.2020.101513

[CR25] Mohammadian M, Fathi-Roudsari M, Mollania N, Badoei-Dalfard A, Khajeh K (2010) Enhanced expression of a recombinant bacterial laccase at low temperature and microaerobic conditions: purification and biochemical characterization. J Ind Microbiol Biotechnol 37(8):863–869. 10.1007/s10295-010-0734-5 20473548 10.1007/s10295-010-0734-5

[CR26] Pawlik A, Wójcik M, Rułka K, Motyl-Gorzel K, Osińska-Jaroszuk M, Wielbo J, Janusz G (2016) Purification and characterization of laccase from *Sinorhizobium meliloti* and analysis of the lacc gene. Int J Biol Macromol 92:138–147. 10.1016/j.ijbiomac.2016.07.012 27392777 10.1016/j.ijbiomac.2016.07.012

[CR27] Rajeeva S, Lele SS (2011) Three-phase partitioning for concentration and purification of laccase produced by submerged cultures of *Ganoderma sp.* WR-1. Biochem Eng J 54(2):103–110. 10.1016/j.bej.2011.02.006

[CR501] Rao JL, Satyanarayana UM, Aool T (2008) Biochem Biotechnol 150:205–219 10.1007/s12010-008-8171-x 18443744

[CR28] Rezaei S, Shahverdi AR, Faramarzi MA (2017) Isolation, one-step affinity purification, and characterization of a polyextremotolerant laccase from the halophilic bacterium *Aquisalibacillus elongatus* and its application in the delignification of sugar beet pulp. Bioresour Technol 230:67–75. 10.1016/j.biortech.2017.01.036 28161622 10.1016/j.biortech.2017.01.036

[CR29] Rodríguez E, Ruiz-Dueñas FJ, Kooistra R, Ram A, Martínez ÁT, Martínez MJ (2008) Isolation of two laccase genes from the white-rot fungus *Pleurotus eryngii* and heterologous expression of the pel3 encoded protein. J Biotechnol 134(1–2):9–19. 10.1016/j.jbiotec.2007.12.008 18291544 10.1016/j.jbiotec.2007.12.008

[CR30] Ruijssenaars HJ, Hartmans S (2004) A cloned *Bacillus halodurans* multicopper oxidase exhibiting alkaline laccase activity. Appl Microbiol Biotechnol 65:177–182. 10.1007/s00253-004-1571-0 15293032 10.1007/s00253-004-1571-0

[CR31] Safary A, Moniri R, Hamzeh-Mivehroud M, Dastmalchi S (2016) A strategy for soluble overexpression and biochemical characterization of halo-thermotolerant *Bacillus* laccase in modified *E. coli*. J Biotechnol 227:56–63. 10.1016/j.jbiotec.2016.04.006 27059481 10.1016/j.jbiotec.2016.04.006

[CR32] Sambasiva Rao KRS, Tripathy NK, Mahalaxmi Y, Prakasham RS (2012) Laccase-and peroxidase-free tyrosinase production by isolated microbial strain. J Microbiol Biotechnol 22(2):207–214. 10.4014/jmb.1106.0603 22370350 10.4014/jmb.1106.06031

[CR603] Saxena L, Iyer BK, Ananthanarayan L (2007) Three phase partitioning as a novel method for purification of ragi (Eleusine coracana) bifunctional amylase/protease inhibitor. Process Biochem 42:491-495. 10.1016/j.procbio.2006.09.016

[CR34] Sondhi S, Sharma P, Saini S, Puri N, Gupta N (2014) Purification and characterization of an extracellular, thermo-alkali-stable, metal tolerant laccase from *Bacillus tequilensis* SN4. PLoS ONE 9(5):e96951. 10.1371/journal.pone.0096951 24871763 10.1371/journal.pone.0096951 PMC4037180

[CR33] Sondhi S, Sharma P, George N, Chauhan PS, Puri N, Gupta N (2015) An extracellular thermo-alkali-stable laccase from *Bacillus tequilensis* SN4, with a potential to biobleach softwood pulp. 3 Biotech 5:175–185. 10.1007/s13205-014-0207-z 28324575 10.1007/s13205-014-0207-z PMC4362739

[CR35] Trubitsina LI, Tishchenko SV, Gabdulkhakov AG, Lisov AV, Zakharova MV, Leontievsky AA (2015) Structural and functional characterization of two-domain laccase from *Streptomyces viridochromogenes*. Biochimie 112:151–159. 10.1016/j.biochi.2015.03.005 25778839 10.1016/j.biochi.2015.03.005

[CR36] Wang J, Feng J, Jia W, Chang S, Li S, Li Y (2015) Lignin engineering through laccase modification: a promising field for energy plant improvement. Biotechnol Biofuels 8:1–11. 10.1186/s13068-015-0331-y 26379777 10.1186/s13068-015-0331-y PMC4570640

[CR37] Yanmış D, Adıgüzel A, Nadaroğlu H, Güllüce M, Demir N (2016) Purification and characterization of laccase from thermophilic *Anoxybacillus gonensis* P39 and its application of removal textile dyes.https://hdl.handle.net/20.500.12809/2502

[CR503] Zhang C, Zhang S, Diao HW (2013) Purification and characterization of a temperature- and ph-stable laccase from the spores of Bacillus vallismortis fmb-103 and its application in the degradation of malachite green. J Agric Food Chem 61(23):5468–5473 10.1021/jf4010498 23706133

[CR600] Zhu L, Gong L, Zhang Y, Wang R, Ge J, Liu Z, Zare RN (2013) Rapid detection of phenol using a membrane containing laccase nanoflowers. Chem–An Asian J 8(10):2358–2360 10.1002/asia.201300020 23423764

[CR39] Zouari-Mechichi H, Mechichi T, Dhouib A, Sayadi S, Martínez AT, Martinez MJ (2006) Laccase purification and characterization from *Trametes trogii* isolated in Tunisia: decolorization of textile dyes by the purified enzyme. Enzym Microb Technol 39(1):141–148. 10.1016/j.enzmictec.2005.11.027

